# (4′-Ferrocenyl-2,2′:6′,2′′-terpyridine-κ^3^ *N* ^1^,*N* ^1′^,*N* ^1′′^)(1,10-phenanthroline-κ^2^ *N*,*N*′)zinc(II) bis­(perchlorate) acetonitrile monosolvate

**DOI:** 10.1107/S1600536809023939

**Published:** 2009-06-27

**Authors:** Si-Ping Tang, Dai-Zhi Kuang, Yong-Lan Feng

**Affiliations:** a Key Laboratory of Functional Organometallic Materials, Hengyang Normal University, Hengyang, Hunan 421008, People’s Republic of China

## Abstract

In the title complex, [FeZn(C_5_H_5_)(C_20_H_14_N_3_)(C_12_H_8_N_2_)](ClO_4_)_2_·CH_3_CN, the Zn^II^ atom is five-coordinated by a tridentate chelating 4′-ferrocenyl-2,2′:6′,2′′-terpyridine (fctpy) ligand and a bidentate chelating 1,10-phenanthroline (phen) ligand in a distorted square-pyramidal environment with a phen N atom located at the apical position [Zn—N = 2.259 (4) Å]. The terpyridyl motif in each fctpy ligand is coplanar, but the cyclo­penta­dienyl ring is twisted by 9.5 (2)° out of coplanarity with each central pyridine. The two cyclo­penta­dienyl rings of the ferrocenyl group are almost eclipsed with a deviation of 4.5 (1)°. In addition, inter­molecular π–π inter­actions [centroid–centroid distance 3.753 (2) Å] are present between the cyclo­penta­dienyl and outer pyridyl rings of the fctpy ligands. One of the perchlorate anions is equally disordered over two positions.

## Related literature

For general background, see: Andres & Schubert (2004 ▶); Barigelletti & Flamigni (2000 ▶); Constable (2007 ▶). For related complexes of the fctpy ligand, see: Aguado *et al.* (2005 ▶); Constable *et al.* (1994 ▶); Farlow *et al.* (1993 ▶); Tang & Kuang (2007 ▶).
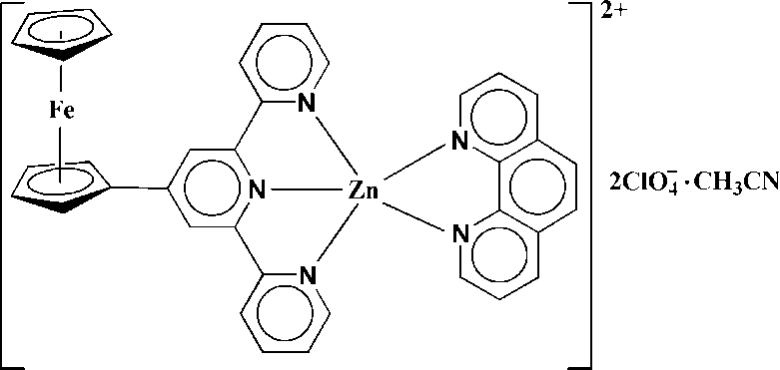


## Experimental

### 

#### Crystal data

[FeZn(C_5_H_5_)(C_20_H_14_N_3_)(C_12_H_8_N_2_)](ClO_4_)_2_·C_2_H_3_N
*M* *_r_* = 902.81
Monoclinic, 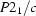

*a* = 13.5426 (12) Å
*b* = 12.0901 (10) Å
*c* = 23.159 (2) Å
β = 97.739 (2)°
*V* = 3757.3 (6) Å^3^
*Z* = 4
Mo *K*α radiation
μ = 1.23 mm^−1^
*T* = 295 K
0.20 × 0.18 × 0.14 mm

#### Data collection

Bruker SMART APEX area-detector diffractometer
Absorption correction: multi-scan (*SADABS*; Sheldrick, 1996 ▶) *T*_min_ = 0.791, *T*_max_ = 0.847
18927 measured reflections
7351 independent reflections
4504 reflections with *I* > 2σ(*I*)
*R*_int_ = 0.042

#### Refinement

*R*[*F* ^2^ > 2σ(*F* ^2^)] = 0.063
*wR*(*F* ^2^) = 0.189
*S* = 1.02
7351 reflections
552 parameters
76 restraints
H-atom parameters constrained
Δρ_max_ = 0.69 e Å^−3^
Δρ_min_ = −0.39 e Å^−3^

### 

Data collection: *SMART* (Bruker, 2002 ▶); cell refinement: *SAINT* (Bruker, 2002 ▶); data reduction: *SAINT*; program(s) used to solve structure: *SHELXS97* (Sheldrick, 2008 ▶); program(s) used to refine structure: *SHELXL97* (Sheldrick, 2008 ▶); molecular graphics: *SHELXTL* (Sheldrick, 2008 ▶); software used to prepare material for publication: *SHELXTL*.

## Supplementary Material

Crystal structure: contains datablocks I, global. DOI: 10.1107/S1600536809023939/jh2081sup1.cif

Structure factors: contains datablocks I. DOI: 10.1107/S1600536809023939/jh2081Isup2.hkl

Additional supplementary materials:  crystallographic information; 3D view; checkCIF report

## Figures and Tables

**Fig. 1.**
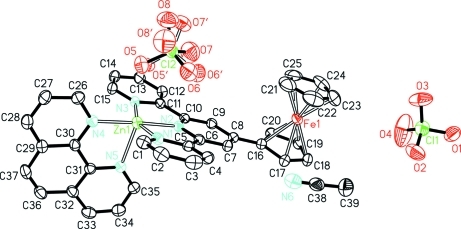
The title molecule with displacement ellipsoids drawn at the 30% probability level, and the H atoms are omitted for clarity.

## supplementary crystallographic information

### Comment

The derivates of 2,2':6',2''-terpyridine (tpy) ligand have attracted wide interests of the chemists because of their good chelating abilities towards transition metal ions, and some of its complexes exhibits potential applications as luminescent devices (Andres & Schubert, 2004; Barigelletti & Flamigni, 2000; Constable, 2007). We and other groups have studied the structures and electrochemical properties of 4'-ferrocenyl-2,2':6',2''- terpyridine (fctpy) and its Au^I^, Ru^II^, Co^II^, Fe^II^ and Cu^II^ complexes (Aguado *et al.*, 2005; Constable *et al.*, 1994; Farlow *et al.*, 1993, Tang & Kuang, 2007). A new mixed-ligand complex, incorporating fctpy and 1,10-phenanthroline (phen) ligands is here presented.

In the title complex, the Zn (II) atom is five-coordinated by three N atoms from the fctpy ligand and two N atoms from the phen ligand, displaying a distorted square pyramidal geometry, as shown in Fig. 1. The apex of the pyramid is occupied by the N5 atom from the phen ligand with a longer Zn—N distance of 2.259 (4) Å than those in the basal plane [Zn—N distances within the range 1.925 (4)-2.057 (4) Å]. The angles subtended at Zn(1) by the terpyridyl motif are 79.61 (16) and 80.30 (15) °, respectively. The terpyridyl motif in each fctpy is fairly coplanar [interplanar dihedral angles 6.7 (2) and 6.3 (2) °], but the cyclopentadienyl ring tilts slightly from its attaching central pyridyl ring of 9.5 (2) °. The two cyclopentadienyl rings of the ferrocenyl group are almost eclipsed with a stagger of 4.5 (1) °, which is smaller than the corresponding value in the similar compound of the title compound (Tang & Kuang, 2007).

In the crystal packing, the cyclopentadienyl rings attached to the central pyridyl ring and the neighboring outer N3-pyridyl rings of the fctpy ligands are involved in intermolecular π–π interactions with the centroid-to-centroid distances of 3.753 (2) Å

### Experimental

A solution of zinc perchlorates hexahydrate (18.6 mg, 0.05 mmol), fctpy (21.0 mg, 0.05 mmol) and 1,10-phenanthroline (9.9 mg, 0.05 mmol) in methanol (10 ml) was stirred for 4 h. The product was filtered off and dried. The precipitate was recrystallized from acetonitrile (5 ml) to give dark-red block-shaped crystals of the title complex after one week. Yield: 20 mg (44%).

### Refinement

One of the perchlorate anions is two-fold disordered and refined with constraints. The carbon-bound H atoms were placed at calculated positions (C—H = 0.93 Å) and refined as riding, with *U*(H) = 1.2*U*_eq_(C) for phenyl and cyclopentadienyl H atoms, and C—H = 0.96 Å and *U*_iso_ = 1.5*U*_eq_ (C) for methyl H atoms.

### Crystal data

**Table:**

[FeZn(C_5_H_5_)(C_20_H_14_N_3_)(C_12_H_8_N_2_)](ClO_4_)_2_·C_2_H_3_N	*F*(000) = 1840
*M**_r_* = 902.81	*D*_x_ = 1.596 Mg m^−^^3^
Monoclinic, *P*2_1_/*c*	Mo *K*α radiation, λ = 0.71073 Å
Hall symbol: -P 2ybc	Cell parameters from 2756 reflections
*a* = 13.5426 (12) Å	θ = 2.3–22.5°
*b* = 12.0901 (10) Å	µ = 1.23 mm^−^^1^
*c* = 23.159 (2) Å	*T* = 295 K
β = 97.739 (2)°	Block, dark red
*V* = 3757.3 (6) Å^3^	0.20 × 0.18 × 0.14 mm
*Z* = 4	

### Data collection

**Table:**

Bruker SMART APEX area-detector diffractometer	7351 independent reflections
Radiation source: fine-focus sealed tube	4504 reflections with *I* > 2σ(*I*)
graphite	*R*_int_ = 0.042
φ and ω scans	θ_max_ = 26.0°, θ_min_ = 1.9°
Absorption correction: multi-scan (*SADABS*; Sheldrick, 1996)	*h* = −16→6
*T*_min_ = 0.791, *T*_max_ = 0.847	*k* = −14→14
18927 measured reflections	*l* = −28→27

### Refinement

**Table:**

Refinement on *F*^2^	Primary atom site location: structure-invariant direct methods
Least-squares matrix: full	Secondary atom site location: difference Fourier map
*R*[*F*^2^ > 2σ(*F*^2^)] = 0.063	Hydrogen site location: inferred from neighbouring sites
*wR*(*F*^2^) = 0.189	H-atom parameters constrained
*S* = 1.02	*w* = 1/[σ^2^(*F*_o_^2^) + (0.0998*P*)^2^ + 0.0603*P*] where *P* = (*F*_o_^2^ + 2*F*_c_^2^)/3
7351 reflections	(Δ/σ)_max_ < 0.001
552 parameters	Δρ_max_ = 0.69 e Å^−^^3^
76 restraints	Δρ_min_ = −0.39 e Å^−^^3^

### Special details

**Table:**

Geometry. All esds (except the esd in the dihedral angle between two l.s. planes) are estimated using the full covariance matrix. The cell esds are taken into account individually in the estimation of esds in distances, angles and torsion angles; correlations between esds in cell parameters are only used when they are defined by crystal symmetry. An approximate (isotropic) treatment of cell esds is used for estimating esds involving l.s. planes.
Refinement. Refinement of F^2^ against ALL reflections. The weighted R-factor wR and goodness of fit S are based on F^2^, conventional R-factors R are based on F, with F set to zero for negative F^2^. The threshold expression of F^2^ > 2sigma(F^2^) is used only for calculating R-factors(gt) etc. and is not relevant to the choice of reflections for refinement. R-factors based on F^2^ are statistically about twice as large as those based on F, and R- factors based on ALL data will be even larger.

### Fractional atomic coordinates and isotropic or equivalent isotropic displacement parameters (Å^2^)

**Table:**

	*x*	*y*	*z*	*U*_iso_*/*U*_eq_	Occ. (<1)
Zn1	0.20436 (4)	0.80770 (5)	0.44899 (2)	0.0548 (2)	
Fe1	0.43574 (6)	0.43885 (6)	0.69019 (3)	0.0600 (3)	
N1	0.0827 (3)	0.7461 (3)	0.48281 (16)	0.0530 (10)	
N2	0.2680 (3)	0.6996 (3)	0.50269 (15)	0.0456 (9)	
N3	0.3505 (3)	0.8502 (3)	0.44757 (15)	0.0487 (9)	
N4	0.1387 (3)	0.9140 (3)	0.38969 (17)	0.0518 (10)	
N5	0.1950 (3)	0.7041 (3)	0.36728 (16)	0.0521 (10)	
C1	−0.0112 (4)	0.7820 (5)	0.4727 (2)	0.0634 (14)	
H1	−0.0263	0.8416	0.4477	0.076*	
C2	−0.0865 (4)	0.7331 (5)	0.4983 (3)	0.0697 (15)	
H2	−0.1513	0.7598	0.4909	0.084*	
C3	−0.0647 (4)	0.6452 (5)	0.5345 (3)	0.0733 (16)	
H3	−0.1148	0.6110	0.5518	0.088*	
C4	0.0323 (4)	0.6068 (4)	0.5454 (2)	0.0617 (14)	
H4	0.0481	0.5470	0.5702	0.074*	
C5	0.1050 (4)	0.6588 (4)	0.5190 (2)	0.0491 (11)	
C6	0.2119 (3)	0.6300 (4)	0.52871 (19)	0.0467 (11)	
C7	0.2552 (4)	0.5428 (4)	0.5613 (2)	0.0508 (12)	
H7	0.2157	0.4935	0.5788	0.061*	
C8	0.3590 (4)	0.5287 (4)	0.56798 (19)	0.0479 (11)	
C9	0.4157 (4)	0.6051 (4)	0.54125 (19)	0.0496 (12)	
H9	0.4848	0.5989	0.5451	0.059*	
C10	0.3671 (4)	0.6908 (4)	0.50863 (19)	0.0458 (11)	
C11	0.4155 (4)	0.7804 (4)	0.47865 (18)	0.0468 (11)	
C12	0.5173 (4)	0.7952 (4)	0.4826 (2)	0.0550 (12)	
H12	0.5608	0.7457	0.5037	0.066*	
C13	0.5535 (4)	0.8837 (5)	0.4551 (2)	0.0630 (14)	
H13	0.6217	0.8961	0.4582	0.076*	
C14	0.4882 (4)	0.9536 (4)	0.4232 (2)	0.0630 (14)	
H14	0.5117	1.0132	0.4035	0.076*	
C15	0.3877 (4)	0.9350 (4)	0.4203 (2)	0.0573 (13)	
H15	0.3438	0.9833	0.3987	0.069*	
C16	0.4066 (4)	0.4362 (4)	0.6018 (2)	0.0538 (12)	
C17	0.3564 (4)	0.3479 (4)	0.6261 (2)	0.0607 (14)	
H17	0.2878	0.3366	0.6214	0.073*	
C18	0.4289 (5)	0.2803 (4)	0.6585 (2)	0.0685 (15)	
H18	0.4163	0.2165	0.6787	0.082*	
C19	0.5223 (5)	0.3254 (4)	0.6551 (2)	0.0627 (14)	
H19	0.5828	0.2968	0.6725	0.075*	
C20	0.5097 (4)	0.4227 (4)	0.6204 (2)	0.0576 (13)	
H20	0.5602	0.4693	0.6116	0.069*	
C21	0.3877 (11)	0.5856 (9)	0.7185 (4)	0.129 (4)	
H21	0.3529	0.6389	0.6950	0.154*	
C22	0.3467 (7)	0.4982 (11)	0.7467 (5)	0.124 (3)	
H22	0.2790	0.4837	0.7456	0.149*	
C23	0.4231 (10)	0.4371 (7)	0.7766 (3)	0.111 (3)	
H23	0.4161	0.3741	0.7988	0.133*	
C24	0.5111 (6)	0.4856 (9)	0.7677 (3)	0.104 (3)	
H24	0.5747	0.4619	0.7827	0.125*	
C25	0.4876 (10)	0.5776 (8)	0.7318 (4)	0.121 (3)	
H25	0.5337	0.6258	0.7190	0.145*	
C26	0.1117 (4)	1.0154 (4)	0.4009 (2)	0.0639 (14)	
H26	0.1209	1.0405	0.4392	0.077*	
C27	0.0695 (4)	1.0866 (5)	0.3568 (3)	0.0670 (15)	
H27	0.0495	1.1572	0.3660	0.080*	
C28	0.0580 (4)	1.0523 (5)	0.3007 (3)	0.0664 (15)	
H28	0.0306	1.0999	0.2713	0.080*	
C29	0.0872 (4)	0.9458 (4)	0.2867 (2)	0.0563 (13)	
C30	0.1280 (3)	0.8781 (4)	0.3332 (2)	0.0495 (12)	
C31	0.1599 (4)	0.7679 (4)	0.3213 (2)	0.0492 (11)	
C32	0.1538 (4)	0.7321 (4)	0.2627 (2)	0.0590 (13)	
C33	0.1874 (4)	0.6231 (5)	0.2546 (3)	0.0736 (16)	
H33	0.1850	0.5943	0.2172	0.088*	
C34	0.2231 (5)	0.5604 (5)	0.3014 (3)	0.0786 (18)	
H34	0.2475	0.4897	0.2962	0.094*	
C35	0.2228 (4)	0.6029 (4)	0.3573 (2)	0.0615 (14)	
H35	0.2434	0.5573	0.3890	0.074*	
C36	0.1118 (5)	0.8027 (5)	0.2176 (2)	0.0738 (17)	
H36	0.1069	0.7788	0.1792	0.089*	
C37	0.0788 (4)	0.9035 (5)	0.2291 (2)	0.0699 (16)	
H37	0.0496	0.9471	0.1984	0.084*	
Cl1	0.29132 (13)	0.20924 (14)	0.88948 (8)	0.0823 (5)	
Cl2	0.20806 (12)	0.95499 (13)	0.58898 (6)	0.0699 (4)	
O1	0.3151 (4)	0.1246 (4)	0.9303 (2)	0.1066 (16)	
O2	0.1957 (5)	0.1830 (4)	0.8569 (2)	0.1237 (19)	
O3	0.2817 (4)	0.3093 (4)	0.9178 (3)	0.131 (2)	
O4	0.3644 (7)	0.2106 (8)	0.8557 (4)	0.239 (5)	
O5	0.1906 (9)	0.9774 (11)	0.5280 (3)	0.097 (4)	0.502 (11)
O6	0.1369 (8)	0.8737 (9)	0.6006 (5)	0.107 (4)	0.502 (11)
O7	0.3013 (7)	0.9012 (11)	0.6063 (5)	0.148 (6)	0.502 (11)
O8	0.2057 (12)	1.0547 (8)	0.6190 (5)	0.139 (5)	0.502 (11)
O8'	0.1199 (8)	1.0109 (11)	0.6002 (6)	0.158 (6)	0.498 (11)
O7'	0.2833 (7)	1.0053 (9)	0.6292 (4)	0.105 (4)	0.498 (11)
O6'	0.1978 (11)	0.8411 (6)	0.6059 (5)	0.113 (4)	0.498 (11)
O5'	0.2334 (8)	0.9585 (9)	0.5319 (3)	0.072 (3)	0.498 (11)
N6	0.0755 (6)	0.3618 (6)	0.6461 (3)	0.109 (2)	
C39	0.1134 (6)	0.2945 (6)	0.6748 (3)	0.085 (2)	
C38	0.1642 (7)	0.2102 (6)	0.7123 (3)	0.115 (3)	
H38A	0.1617	0.1410	0.6917	0.173*	
H38B	0.1321	0.2023	0.7465	0.173*	
H38C	0.2325	0.2314	0.7233	0.173*	

### Atomic displacement parameters (Å^2^)

**Table:**

	*U*^11^	*U*^22^	*U*^33^	*U*^12^	*U*^13^	*U*^23^
Zn1	0.0575 (4)	0.0587 (4)	0.0477 (4)	0.0035 (3)	0.0053 (3)	0.0062 (3)
Fe1	0.0739 (6)	0.0603 (5)	0.0454 (4)	0.0030 (4)	0.0072 (4)	0.0003 (3)
N1	0.052 (2)	0.062 (3)	0.045 (2)	−0.002 (2)	0.0067 (19)	−0.0031 (19)
N2	0.048 (2)	0.047 (2)	0.041 (2)	−0.0035 (18)	0.0045 (17)	0.0043 (17)
N3	0.057 (2)	0.050 (2)	0.038 (2)	−0.002 (2)	0.0055 (18)	0.0031 (17)
N4	0.051 (2)	0.055 (2)	0.049 (2)	0.006 (2)	0.0072 (19)	−0.0014 (19)
N5	0.056 (2)	0.052 (2)	0.047 (2)	−0.001 (2)	0.0021 (19)	0.0006 (19)
C1	0.058 (3)	0.070 (3)	0.064 (3)	0.003 (3)	0.012 (3)	−0.007 (3)
C2	0.053 (3)	0.086 (4)	0.070 (4)	0.012 (3)	0.008 (3)	−0.013 (3)
C3	0.056 (3)	0.085 (4)	0.085 (4)	−0.011 (3)	0.027 (3)	−0.020 (4)
C4	0.071 (4)	0.057 (3)	0.059 (3)	−0.008 (3)	0.014 (3)	−0.003 (3)
C5	0.057 (3)	0.046 (3)	0.045 (3)	−0.006 (2)	0.009 (2)	−0.006 (2)
C6	0.049 (3)	0.054 (3)	0.038 (2)	−0.004 (2)	0.008 (2)	−0.003 (2)
C7	0.059 (3)	0.048 (3)	0.047 (3)	−0.006 (2)	0.012 (2)	0.006 (2)
C8	0.059 (3)	0.045 (3)	0.040 (2)	−0.003 (2)	0.007 (2)	0.000 (2)
C9	0.046 (3)	0.054 (3)	0.047 (3)	−0.005 (2)	0.002 (2)	0.001 (2)
C10	0.051 (3)	0.049 (3)	0.037 (2)	−0.006 (2)	0.003 (2)	−0.002 (2)
C11	0.053 (3)	0.050 (3)	0.038 (2)	0.001 (2)	0.007 (2)	−0.001 (2)
C12	0.054 (3)	0.060 (3)	0.051 (3)	0.002 (3)	0.008 (2)	0.006 (2)
C13	0.061 (3)	0.075 (4)	0.054 (3)	−0.016 (3)	0.014 (3)	0.004 (3)
C14	0.076 (4)	0.060 (3)	0.055 (3)	−0.014 (3)	0.019 (3)	0.010 (3)
C15	0.071 (4)	0.050 (3)	0.051 (3)	0.000 (3)	0.010 (3)	0.012 (2)
C16	0.067 (3)	0.048 (3)	0.047 (3)	−0.003 (2)	0.007 (2)	0.001 (2)
C17	0.071 (4)	0.053 (3)	0.058 (3)	−0.008 (3)	0.007 (3)	0.009 (2)
C18	0.093 (4)	0.054 (3)	0.058 (3)	0.004 (3)	0.009 (3)	0.014 (3)
C19	0.073 (4)	0.056 (3)	0.059 (3)	0.016 (3)	0.007 (3)	0.004 (2)
C20	0.071 (4)	0.048 (3)	0.053 (3)	0.004 (3)	0.005 (3)	0.001 (2)
C21	0.188 (11)	0.107 (7)	0.085 (6)	0.044 (8)	−0.001 (8)	−0.031 (5)
C22	0.102 (7)	0.167 (10)	0.112 (7)	−0.004 (7)	0.046 (6)	−0.060 (7)
C23	0.168 (9)	0.114 (6)	0.057 (4)	−0.006 (7)	0.035 (5)	−0.002 (4)
C24	0.095 (6)	0.154 (8)	0.058 (4)	0.024 (6)	−0.013 (4)	−0.028 (5)
C25	0.182 (10)	0.104 (6)	0.081 (6)	−0.055 (7)	0.031 (7)	−0.046 (5)
C26	0.068 (3)	0.056 (3)	0.068 (3)	0.007 (3)	0.010 (3)	−0.002 (3)
C27	0.062 (3)	0.057 (3)	0.081 (4)	0.013 (3)	0.007 (3)	0.008 (3)
C28	0.059 (3)	0.071 (4)	0.067 (4)	0.003 (3)	0.002 (3)	0.016 (3)
C29	0.057 (3)	0.060 (3)	0.052 (3)	0.002 (3)	0.006 (2)	0.008 (2)
C30	0.042 (3)	0.056 (3)	0.049 (3)	−0.005 (2)	0.000 (2)	0.008 (2)
C31	0.048 (3)	0.049 (3)	0.050 (3)	−0.001 (2)	0.006 (2)	−0.001 (2)
C32	0.064 (3)	0.069 (3)	0.044 (3)	−0.005 (3)	0.007 (2)	−0.001 (2)
C33	0.086 (4)	0.077 (4)	0.059 (4)	0.000 (3)	0.015 (3)	−0.022 (3)
C34	0.101 (5)	0.060 (3)	0.074 (4)	0.002 (3)	0.011 (4)	−0.011 (3)
C35	0.071 (4)	0.051 (3)	0.061 (3)	0.004 (3)	0.005 (3)	−0.002 (2)
C36	0.088 (4)	0.086 (4)	0.045 (3)	−0.009 (4)	0.002 (3)	−0.002 (3)
C37	0.078 (4)	0.084 (4)	0.045 (3)	0.002 (3)	0.000 (3)	0.014 (3)
Cl1	0.0835 (11)	0.0773 (10)	0.0883 (11)	0.0114 (9)	0.0201 (9)	0.0003 (9)
Cl2	0.0786 (10)	0.0795 (10)	0.0535 (8)	−0.0053 (8)	0.0158 (7)	−0.0020 (7)
O1	0.110 (4)	0.079 (3)	0.122 (4)	−0.010 (3)	−0.017 (3)	0.013 (3)
O2	0.138 (5)	0.129 (4)	0.092 (3)	0.022 (4)	−0.028 (3)	−0.025 (3)
O3	0.114 (4)	0.079 (3)	0.186 (6)	0.019 (3)	−0.038 (4)	−0.044 (3)
O4	0.218 (8)	0.266 (10)	0.274 (9)	0.077 (8)	0.186 (8)	0.118 (8)
O5	0.105 (7)	0.109 (6)	0.076 (5)	0.011 (5)	0.007 (4)	0.018 (4)
O6	0.092 (6)	0.138 (6)	0.096 (6)	−0.028 (5)	0.036 (5)	0.005 (5)
O7	0.136 (7)	0.161 (7)	0.143 (7)	0.014 (5)	0.002 (5)	0.008 (5)
O8	0.161 (7)	0.127 (6)	0.129 (7)	0.013 (5)	0.013 (5)	−0.044 (5)
O8'	0.130 (7)	0.172 (8)	0.175 (8)	0.021 (5)	0.032 (5)	0.004 (5)
O7'	0.100 (6)	0.130 (6)	0.083 (5)	−0.022 (5)	0.002 (4)	−0.014 (4)
O6'	0.143 (7)	0.099 (6)	0.097 (6)	0.007 (5)	0.019 (5)	0.014 (4)
O5'	0.083 (5)	0.082 (5)	0.055 (4)	−0.016 (4)	0.023 (4)	−0.007 (4)
N6	0.135 (6)	0.103 (5)	0.086 (5)	−0.011 (4)	0.009 (4)	−0.021 (4)
C39	0.110 (6)	0.087 (5)	0.063 (4)	−0.021 (4)	0.029 (4)	−0.021 (4)
C38	0.140 (7)	0.113 (6)	0.096 (5)	−0.010 (6)	0.029 (5)	0.014 (5)

### Geometric parameters (Å, °)

**Table:**

Zn1—N2	1.925 (4)	C17—H17	0.9300
Zn1—N4	2.000 (4)	C18—C19	1.390 (8)
Zn1—N3	2.050 (4)	C18—H18	0.9300
Zn1—N1	2.057 (4)	C19—C20	1.421 (7)
Zn1—N5	2.259 (4)	C19—H19	0.9300
Fe1—C25	2.013 (7)	C20—H20	0.9300
Fe1—C20	2.021 (5)	C21—C25	1.349 (13)
Fe1—C24	2.022 (6)	C21—C22	1.397 (13)
Fe1—C22	2.027 (8)	C21—H21	0.9300
Fe1—C21	2.028 (8)	C22—C23	1.380 (12)
Fe1—C23	2.031 (7)	C22—H22	0.9300
Fe1—C16	2.031 (5)	C23—C24	1.368 (11)
Fe1—C17	2.034 (5)	C23—H23	0.9300
Fe1—C19	2.043 (5)	C24—C25	1.400 (11)
Fe1—C18	2.050 (6)	C24—H24	0.9300
N1—C1	1.334 (6)	C25—H25	0.9300
N1—C5	1.356 (6)	C26—C27	1.396 (7)
N2—C6	1.331 (6)	C26—H26	0.9300
N2—C10	1.335 (6)	C27—C28	1.354 (8)
N3—C15	1.337 (6)	C27—H27	0.9300
N3—C11	1.355 (6)	C28—C29	1.398 (7)
N4—C26	1.316 (6)	C28—H28	0.9300
N4—C30	1.366 (6)	C29—C30	1.407 (6)
N5—C35	1.310 (6)	C29—C37	1.419 (6)
N5—C31	1.350 (6)	C30—C31	1.439 (7)
C1—C2	1.379 (8)	C31—C32	1.415 (6)
C1—H1	0.9300	C32—C36	1.409 (6)
C2—C3	1.361 (8)	C32—C33	1.416 (8)
C2—H2	0.9300	C33—C34	1.357 (8)
C3—C4	1.384 (8)	C33—H33	0.9300
C3—H3	0.9300	C34—C35	1.393 (8)
C4—C5	1.380 (7)	C34—H34	0.9300
C4—H4	0.9300	C35—H35	0.9300
C5—C6	1.476 (7)	C36—C37	1.336 (7)
C6—C7	1.380 (6)	C36—H36	0.9300
C7—C8	1.404 (7)	C37—H37	0.9300
C7—H7	0.9300	Cl1—O4	1.342 (7)
C8—C9	1.398 (6)	Cl1—O3	1.391 (5)
C8—C16	1.465 (6)	Cl1—O1	1.400 (5)
C9—C10	1.395 (6)	Cl1—O2	1.443 (5)
C9—H9	0.9300	Cl2—O8	1.393 (7)
C10—C11	1.486 (6)	Cl2—O5'	1.411 (6)
C11—C12	1.381 (7)	Cl2—O7'	1.421 (7)
C12—C13	1.369 (7)	Cl2—O8'	1.426 (8)
C12—H12	0.9300	Cl2—O6	1.427 (7)
C13—C14	1.367 (7)	Cl2—O5	1.427 (7)
C13—H13	0.9300	Cl2—O7	1.429 (8)
C14—C15	1.373 (7)	Cl2—O6'	1.443 (7)
C14—H14	0.9300	N6—C39	1.129 (9)
C15—H15	0.9300	C39—C38	1.451 (10)
C16—C20	1.414 (7)	C38—H38A	0.9600
C16—C17	1.421 (7)	C38—H38B	0.9600
C17—C18	1.414 (7)	C38—H38C	0.9600
			
N2—Zn1—N4	176.86 (16)	C16—C17—H17	126.0
N2—Zn1—N3	80.30 (15)	Fe1—C17—H17	125.8
N4—Zn1—N3	99.75 (15)	C19—C18—C17	108.3 (5)
N2—Zn1—N1	79.61 (16)	C19—C18—Fe1	69.9 (3)
N4—Zn1—N1	100.90 (16)	C17—C18—Fe1	69.1 (3)
N3—Zn1—N1	157.18 (15)	C19—C18—H18	125.8
N2—Zn1—N5	97.73 (15)	C17—C18—H18	125.8
N4—Zn1—N5	79.13 (15)	Fe1—C18—H18	126.7
N3—Zn1—N5	94.09 (15)	C18—C19—C20	108.5 (5)
N1—Zn1—N5	99.23 (15)	C18—C19—Fe1	70.4 (3)
C25—Fe1—C20	106.5 (3)	C20—C19—Fe1	68.7 (3)
C25—Fe1—C24	40.6 (3)	C18—C19—H19	125.8
C20—Fe1—C24	119.4 (3)	C20—C19—H19	125.8
C25—Fe1—C22	66.1 (4)	Fe1—C19—H19	126.6
C20—Fe1—C22	162.2 (5)	C16—C20—C19	107.8 (5)
C24—Fe1—C22	66.5 (3)	C16—C20—Fe1	69.9 (3)
C25—Fe1—C21	39.0 (4)	C19—C20—Fe1	70.3 (3)
C20—Fe1—C21	123.9 (4)	C16—C20—H20	126.1
C24—Fe1—C21	67.3 (4)	C19—C20—H20	126.1
C22—Fe1—C21	40.3 (4)	Fe1—C20—H20	125.2
C25—Fe1—C23	66.9 (3)	C25—C21—C22	106.8 (9)
C20—Fe1—C23	154.5 (4)	C25—C21—Fe1	69.9 (5)
C24—Fe1—C23	39.5 (3)	C22—C21—Fe1	69.8 (5)
C22—Fe1—C23	39.7 (4)	C25—C21—H21	126.6
C21—Fe1—C23	67.5 (4)	C22—C21—H21	126.6
C25—Fe1—C16	120.2 (3)	Fe1—C21—H21	125.3
C20—Fe1—C16	40.9 (2)	C23—C22—C21	108.7 (9)
C24—Fe1—C16	154.8 (4)	C23—C22—Fe1	70.3 (5)
C22—Fe1—C16	127.2 (4)	C21—C22—Fe1	69.9 (5)
C21—Fe1—C16	108.2 (3)	C23—C22—H22	125.6
C23—Fe1—C16	164.0 (4)	C21—C22—H22	125.6
C25—Fe1—C17	156.3 (4)	Fe1—C22—H22	125.8
C20—Fe1—C17	68.6 (2)	C24—C23—C22	107.7 (8)
C24—Fe1—C17	162.3 (4)	C24—C23—Fe1	69.9 (4)
C22—Fe1—C17	111.2 (3)	C22—C23—Fe1	70.0 (4)
C21—Fe1—C17	123.2 (4)	C24—C23—H23	126.1
C23—Fe1—C17	127.4 (3)	C22—C23—H23	126.1
C16—Fe1—C17	40.90 (19)	Fe1—C23—H23	125.5
C25—Fe1—C19	124.6 (4)	C23—C24—C25	107.3 (8)
C20—Fe1—C19	40.93 (19)	C23—C24—Fe1	70.6 (4)
C24—Fe1—C19	107.0 (3)	C25—C24—Fe1	69.3 (4)
C22—Fe1—C19	156.6 (5)	C23—C24—H24	126.3
C21—Fe1—C19	160.4 (5)	C25—C24—H24	126.3
C23—Fe1—C19	120.9 (4)	Fe1—C24—H24	125.3
C16—Fe1—C19	68.4 (2)	C21—C25—C24	109.4 (9)
C17—Fe1—C19	67.8 (2)	C21—C25—Fe1	71.1 (5)
C25—Fe1—C18	161.1 (5)	C24—C25—Fe1	70.1 (4)
C20—Fe1—C18	68.1 (2)	C21—C25—H25	125.3
C24—Fe1—C18	124.9 (3)	C24—C25—H25	125.3
C22—Fe1—C18	124.1 (4)	Fe1—C25—H25	125.2
C21—Fe1—C18	158.8 (5)	N4—C26—C27	121.9 (5)
C23—Fe1—C18	109.7 (3)	N4—C26—H26	119.1
C16—Fe1—C18	68.38 (19)	C27—C26—H26	119.1
C17—Fe1—C18	40.5 (2)	C28—C27—C26	119.8 (5)
C19—Fe1—C18	39.7 (2)	C28—C27—H27	120.1
C1—N1—C5	119.3 (5)	C26—C27—H27	120.1
C1—N1—Zn1	127.5 (4)	C27—C28—C29	120.3 (5)
C5—N1—Zn1	113.2 (3)	C27—C28—H28	119.8
C6—N2—C10	122.0 (4)	C29—C28—H28	119.8
C6—N2—Zn1	119.3 (3)	C28—C29—C30	116.9 (5)
C10—N2—Zn1	118.5 (3)	C28—C29—C37	124.2 (5)
C15—N3—C11	118.0 (4)	C30—C29—C37	119.0 (5)
C15—N3—Zn1	128.7 (3)	N4—C30—C29	122.0 (4)
C11—N3—Zn1	113.3 (3)	N4—C30—C31	118.8 (4)
C26—N4—C30	119.2 (4)	C29—C30—C31	119.2 (4)
C26—N4—Zn1	125.0 (4)	N5—C31—C32	123.4 (4)
C30—N4—Zn1	115.7 (3)	N5—C31—C30	117.4 (4)
C35—N5—C31	118.3 (4)	C32—C31—C30	119.2 (4)
C35—N5—Zn1	132.9 (3)	C36—C32—C31	119.4 (5)
C31—N5—Zn1	108.6 (3)	C36—C32—C33	124.8 (5)
N1—C1—C2	121.8 (5)	C31—C32—C33	115.7 (4)
N1—C1—H1	119.1	C34—C33—C32	120.0 (5)
C2—C1—H1	119.1	C34—C33—H33	120.0
C3—C2—C1	119.2 (5)	C32—C33—H33	120.0
C3—C2—H2	120.4	C33—C34—C35	119.4 (5)
C1—C2—H2	120.4	C33—C34—H34	120.3
C2—C3—C4	119.8 (6)	C35—C34—H34	120.3
C2—C3—H3	120.1	N5—C35—C34	123.0 (5)
C4—C3—H3	120.1	N5—C35—H35	118.5
C5—C4—C3	118.8 (5)	C34—C35—H35	118.5
C5—C4—H4	120.6	C37—C36—C32	121.2 (5)
C3—C4—H4	120.6	C37—C36—H36	119.4
N1—C5—C4	121.1 (5)	C32—C36—H36	119.4
N1—C5—C6	114.2 (4)	C36—C37—C29	122.0 (5)
C4—C5—C6	124.7 (4)	C36—C37—H37	119.0
N2—C6—C7	120.3 (4)	C29—C37—H37	119.0
N2—C6—C5	112.9 (4)	O4—Cl1—O3	112.8 (6)
C7—C6—C5	126.8 (4)	O4—Cl1—O1	106.3 (4)
C6—C7—C8	119.9 (4)	O3—Cl1—O1	110.2 (3)
C6—C7—H7	120.1	O4—Cl1—O2	112.2 (6)
C8—C7—H7	120.1	O3—Cl1—O2	107.7 (3)
C9—C8—C7	118.2 (4)	O1—Cl1—O2	107.5 (3)
C9—C8—C16	120.9 (4)	O8—Cl2—O5'	117.8 (7)
C7—C8—C16	120.9 (4)	O8—Cl2—O7'	50.7 (6)
C10—C9—C8	118.9 (4)	O5'—Cl2—O7'	111.0 (6)
C10—C9—H9	120.5	O8—Cl2—O8'	54.7 (7)
C8—C9—H9	120.5	O5'—Cl2—O8'	118.1 (7)
N2—C10—C9	120.7 (4)	O7'—Cl2—O8'	102.8 (7)
N2—C10—C11	113.3 (4)	O8—Cl2—O6	116.1 (7)
C9—C10—C11	126.1 (4)	O5'—Cl2—O6	116.9 (6)
N3—C11—C12	121.7 (4)	O7'—Cl2—O6	127.8 (6)
N3—C11—C10	114.0 (4)	O8'—Cl2—O6	71.9 (7)
C12—C11—C10	124.3 (4)	O8—Cl2—O5	108.5 (7)
C13—C12—C11	119.1 (5)	O7'—Cl2—O5	125.2 (7)
C13—C12—H12	120.4	O8'—Cl2—O5	93.4 (7)
C11—C12—H12	120.4	O6—Cl2—O5	107.0 (7)
C14—C13—C12	119.4 (5)	O8—Cl2—O7	109.4 (7)
C14—C13—H13	120.3	O5'—Cl2—O7	87.7 (7)
C12—C13—H13	120.3	O7'—Cl2—O7	58.8 (6)
C13—C14—C15	119.3 (5)	O8'—Cl2—O7	153.4 (7)
C13—C14—H14	120.4	O6—Cl2—O7	103.1 (7)
C15—C14—H14	120.4	O5—Cl2—O7	112.8 (7)
N3—C15—C14	122.5 (5)	O8—Cl2—O6'	132.9 (7)
N3—C15—H15	118.8	O5'—Cl2—O6'	109.1 (6)
C14—C15—H15	118.8	O7'—Cl2—O6'	108.5 (6)
C20—C16—C17	107.4 (4)	O8'—Cl2—O6'	106.8 (7)
C20—C16—C8	126.5 (5)	O5—Cl2—O6'	116.2 (7)
C17—C16—C8	125.9 (5)	O7—Cl2—O6'	66.7 (7)
C20—C16—Fe1	69.2 (3)	N6—C39—C38	178.4 (9)
C17—C16—Fe1	69.7 (3)	C39—C38—H38A	109.5
C8—C16—Fe1	122.6 (3)	C39—C38—H38B	109.5
C18—C17—C16	108.0 (5)	H38A—C38—H38B	109.5
C18—C17—Fe1	70.3 (3)	C39—C38—H38C	109.5
C16—C17—Fe1	69.4 (3)	H38A—C38—H38C	109.5
C18—C17—H17	126.0	H38B—C38—H38C	109.5
			
N2—Zn1—N1—C1	174.4 (4)	C25—Fe1—C19—C20	−74.4 (5)
N4—Zn1—N1—C1	−8.7 (4)	C24—Fe1—C19—C20	−115.5 (5)
N3—Zn1—N1—C1	145.7 (4)	C22—Fe1—C19—C20	174.6 (7)
N5—Zn1—N1—C1	−89.3 (4)	C21—Fe1—C19—C20	−45.5 (10)
N2—Zn1—N1—C5	−5.9 (3)	C23—Fe1—C19—C20	−156.3 (5)
N4—Zn1—N1—C5	170.9 (3)	C16—Fe1—C19—C20	38.2 (3)
N3—Zn1—N1—C5	−34.6 (6)	C17—Fe1—C19—C20	82.4 (3)
N5—Zn1—N1—C5	90.3 (3)	C18—Fe1—C19—C20	119.9 (5)
N3—Zn1—N2—C6	177.7 (3)	C17—C16—C20—C19	1.0 (6)
N1—Zn1—N2—C6	8.6 (3)	C8—C16—C20—C19	176.3 (4)
N5—Zn1—N2—C6	−89.4 (3)	Fe1—C16—C20—C19	60.4 (3)
N3—Zn1—N2—C10	−8.1 (3)	C17—C16—C20—Fe1	−59.4 (3)
N1—Zn1—N2—C10	−177.2 (4)	C8—C16—C20—Fe1	115.8 (5)
N5—Zn1—N2—C10	84.8 (3)	C18—C19—C20—C16	−0.8 (6)
N2—Zn1—N3—C15	−175.2 (4)	Fe1—C19—C20—C16	−60.2 (4)
N4—Zn1—N3—C15	8.0 (4)	C18—C19—C20—Fe1	59.4 (4)
N1—Zn1—N3—C15	−146.6 (4)	C25—Fe1—C20—C16	−117.4 (5)
N5—Zn1—N3—C15	87.7 (4)	C24—Fe1—C20—C16	−159.5 (4)
N2—Zn1—N3—C11	5.2 (3)	C22—Fe1—C20—C16	−54.6 (11)
N4—Zn1—N3—C11	−171.6 (3)	C21—Fe1—C20—C16	−78.4 (5)
N1—Zn1—N3—C11	33.8 (6)	C23—Fe1—C20—C16	171.8 (6)
N5—Zn1—N3—C11	−91.9 (3)	C17—Fe1—C20—C16	38.1 (3)
N3—Zn1—N4—C26	−87.6 (4)	C19—Fe1—C20—C16	118.4 (4)
N1—Zn1—N4—C26	82.6 (4)	C18—Fe1—C20—C16	81.8 (3)
N5—Zn1—N4—C26	−180.0 (5)	C25—Fe1—C20—C19	124.2 (5)
N3—Zn1—N4—C30	88.0 (3)	C24—Fe1—C20—C19	82.1 (5)
N1—Zn1—N4—C30	−101.8 (3)	C22—Fe1—C20—C19	−173.0 (10)
N5—Zn1—N4—C30	−4.4 (3)	C21—Fe1—C20—C19	163.2 (5)
N2—Zn1—N5—C35	−0.1 (5)	C23—Fe1—C20—C19	53.4 (8)
N4—Zn1—N5—C35	179.8 (5)	C16—Fe1—C20—C19	−118.4 (4)
N3—Zn1—N5—C35	80.6 (5)	C17—Fe1—C20—C19	−80.4 (3)
N1—Zn1—N5—C35	−80.8 (5)	C18—Fe1—C20—C19	−36.6 (3)
N2—Zn1—N5—C31	−174.4 (3)	C20—Fe1—C21—C25	−73.5 (7)
N4—Zn1—N5—C31	5.6 (3)	C24—Fe1—C21—C25	37.7 (6)
N3—Zn1—N5—C31	−93.6 (3)	C22—Fe1—C21—C25	117.5 (9)
N1—Zn1—N5—C31	105.0 (3)	C23—Fe1—C21—C25	80.6 (6)
C5—N1—C1—C2	0.3 (7)	C16—Fe1—C21—C25	−115.9 (6)
Zn1—N1—C1—C2	180.0 (4)	C17—Fe1—C21—C25	−158.5 (5)
N1—C1—C2—C3	−0.6 (8)	C19—Fe1—C21—C25	−39.2 (13)
C1—C2—C3—C4	0.5 (8)	C18—Fe1—C21—C25	167.2 (7)
C2—C3—C4—C5	−0.3 (8)	C25—Fe1—C21—C22	−117.5 (9)
C1—N1—C5—C4	−0.1 (7)	C20—Fe1—C21—C22	169.0 (6)
Zn1—N1—C5—C4	−179.8 (4)	C24—Fe1—C21—C22	−79.8 (6)
C1—N1—C5—C6	−177.4 (4)	C23—Fe1—C21—C22	−37.0 (6)
Zn1—N1—C5—C6	2.9 (5)	C16—Fe1—C21—C22	126.6 (6)
C3—C4—C5—N1	0.1 (7)	C17—Fe1—C21—C22	84.0 (7)
C3—C4—C5—C6	177.1 (5)	C19—Fe1—C21—C22	−156.7 (8)
C10—N2—C6—C7	−2.5 (7)	C18—Fe1—C21—C22	49.7 (11)
Zn1—N2—C6—C7	171.5 (3)	C25—C21—C22—C23	−0.7 (10)
C10—N2—C6—C5	176.7 (4)	Fe1—C21—C22—C23	59.8 (6)
Zn1—N2—C6—C5	−9.3 (5)	C25—C21—C22—Fe1	−60.4 (6)
N1—C5—C6—N2	3.7 (6)	C25—Fe1—C22—C23	−82.0 (6)
C4—C5—C6—N2	−173.5 (4)	C20—Fe1—C22—C23	−150.8 (9)
N1—C5—C6—C7	−177.2 (4)	C24—Fe1—C22—C23	−37.5 (5)
C4—C5—C6—C7	5.6 (8)	C21—Fe1—C22—C23	−119.6 (8)
N2—C6—C7—C8	1.2 (7)	C16—Fe1—C22—C23	167.2 (5)
C5—C6—C7—C8	−177.9 (4)	C17—Fe1—C22—C23	123.5 (6)
C6—C7—C8—C9	0.4 (7)	C19—Fe1—C22—C23	40.8 (11)
C6—C7—C8—C16	−179.3 (4)	C18—Fe1—C22—C23	79.8 (6)
C7—C8—C9—C10	−0.8 (6)	C25—Fe1—C22—C21	37.6 (6)
C16—C8—C9—C10	178.9 (4)	C20—Fe1—C22—C21	−31.2 (14)
C6—N2—C10—C9	2.1 (7)	C24—Fe1—C22—C21	82.1 (6)
Zn1—N2—C10—C9	−172.0 (3)	C23—Fe1—C22—C21	119.6 (8)
C6—N2—C10—C11	−176.8 (4)	C16—Fe1—C22—C21	−73.2 (7)
Zn1—N2—C10—C11	9.1 (5)	C17—Fe1—C22—C21	−116.8 (6)
C8—C9—C10—N2	−0.4 (7)	C19—Fe1—C22—C21	160.4 (8)
C8—C9—C10—C11	178.4 (4)	C18—Fe1—C22—C21	−160.6 (6)
C15—N3—C11—C12	0.1 (6)	C21—C22—C23—C24	0.5 (9)
Zn1—N3—C11—C12	179.7 (4)	Fe1—C22—C23—C24	60.0 (5)
C15—N3—C11—C10	178.3 (4)	C21—C22—C23—Fe1	−59.5 (6)
Zn1—N3—C11—C10	−2.0 (5)	C25—Fe1—C23—C24	−38.6 (6)
N2—C10—C11—N3	−4.2 (5)	C20—Fe1—C23—C24	41.2 (10)
C9—C10—C11—N3	176.9 (4)	C22—Fe1—C23—C24	−118.6 (8)
N2—C10—C11—C12	174.0 (4)	C21—Fe1—C23—C24	−81.1 (6)
C9—C10—C11—C12	−4.8 (7)	C16—Fe1—C23—C24	−158.5 (9)
N3—C11—C12—C13	1.0 (7)	C17—Fe1—C23—C24	163.4 (5)
C10—C11—C12—C13	−177.1 (4)	C19—Fe1—C23—C24	79.0 (6)
C11—C12—C13—C14	−1.7 (8)	C18—Fe1—C23—C24	121.4 (6)
C12—C13—C14—C15	1.4 (8)	C25—Fe1—C23—C22	79.9 (6)
C11—N3—C15—C14	−0.4 (7)	C20—Fe1—C23—C22	159.7 (7)
Zn1—N3—C15—C14	−179.9 (4)	C24—Fe1—C23—C22	118.6 (8)
C13—C14—C15—N3	−0.4 (8)	C21—Fe1—C23—C22	37.5 (6)
C9—C8—C16—C20	12.0 (7)	C16—Fe1—C23—C22	−40.0 (13)
C7—C8—C16—C20	−168.2 (5)	C17—Fe1—C23—C22	−78.0 (7)
C9—C8—C16—C17	−173.5 (5)	C19—Fe1—C23—C22	−162.4 (6)
C7—C8—C16—C17	6.2 (7)	C18—Fe1—C23—C22	−120.0 (6)
C9—C8—C16—Fe1	99.1 (5)	C22—C23—C24—C25	−0.1 (9)
C7—C8—C16—Fe1	−81.1 (5)	Fe1—C23—C24—C25	59.9 (5)
C25—Fe1—C16—C20	80.2 (5)	C22—C23—C24—Fe1	−60.0 (5)
C24—Fe1—C16—C20	46.0 (7)	C25—Fe1—C24—C23	118.0 (8)
C22—Fe1—C16—C20	161.8 (5)	C20—Fe1—C24—C23	−161.0 (5)
C21—Fe1—C16—C20	121.1 (6)	C22—Fe1—C24—C23	37.8 (6)
C23—Fe1—C16—C20	−167.2 (10)	C21—Fe1—C24—C23	81.8 (6)
C17—Fe1—C16—C20	−118.8 (4)	C16—Fe1—C24—C23	166.3 (6)
C19—Fe1—C16—C20	−38.3 (3)	C17—Fe1—C24—C23	−48.2 (11)
C18—Fe1—C16—C20	−81.1 (3)	C19—Fe1—C24—C23	−118.3 (6)
C25—Fe1—C16—C17	−161.0 (5)	C18—Fe1—C24—C23	−78.4 (6)
C20—Fe1—C16—C17	118.8 (4)	C20—Fe1—C24—C25	81.0 (7)
C24—Fe1—C16—C17	164.7 (6)	C22—Fe1—C24—C25	−80.2 (7)
C22—Fe1—C16—C17	−79.4 (6)	C21—Fe1—C24—C25	−36.3 (6)
C21—Fe1—C16—C17	−120.1 (6)	C23—Fe1—C24—C25	−118.0 (8)
C23—Fe1—C16—C17	−48.4 (11)	C16—Fe1—C24—C25	48.3 (9)
C19—Fe1—C16—C17	80.5 (3)	C17—Fe1—C24—C25	−166.2 (8)
C18—Fe1—C16—C17	37.7 (3)	C19—Fe1—C24—C25	123.7 (6)
C25—Fe1—C16—C8	−40.7 (7)	C18—Fe1—C24—C25	163.6 (6)
C20—Fe1—C16—C8	−120.9 (5)	C22—C21—C25—C24	0.6 (9)
C24—Fe1—C16—C8	−74.9 (8)	Fe1—C21—C25—C24	−59.8 (5)
C22—Fe1—C16—C8	40.9 (7)	C22—C21—C25—Fe1	60.4 (6)
C21—Fe1—C16—C8	0.3 (7)	C23—C24—C25—C21	−0.3 (9)
C23—Fe1—C16—C8	72.0 (11)	Fe1—C24—C25—C21	60.4 (6)
C17—Fe1—C16—C8	120.3 (6)	C23—C24—C25—Fe1	−60.7 (5)
C19—Fe1—C16—C8	−159.1 (5)	C20—Fe1—C25—C21	123.9 (7)
C18—Fe1—C16—C8	158.0 (5)	C24—Fe1—C25—C21	−119.9 (9)
C20—C16—C17—C18	−0.9 (6)	C22—Fe1—C25—C21	−38.8 (6)
C8—C16—C17—C18	−176.2 (5)	C23—Fe1—C25—C21	−82.3 (7)
Fe1—C16—C17—C18	−60.0 (4)	C16—Fe1—C25—C21	81.6 (7)
C20—C16—C17—Fe1	59.1 (3)	C17—Fe1—C25—C21	49.7 (11)
C8—C16—C17—Fe1	−116.2 (5)	C19—Fe1—C25—C21	165.0 (6)
C25—Fe1—C17—C18	163.2 (8)	C18—Fe1—C25—C21	−165.7 (8)
C20—Fe1—C17—C18	81.0 (4)	C20—Fe1—C25—C24	−116.2 (6)
C24—Fe1—C17—C18	−39.4 (10)	C22—Fe1—C25—C24	81.1 (6)
C22—Fe1—C17—C18	−118.1 (6)	C21—Fe1—C25—C24	119.9 (9)
C21—Fe1—C17—C18	−161.7 (6)	C23—Fe1—C25—C24	37.6 (5)
C23—Fe1—C17—C18	−76.0 (6)	C16—Fe1—C25—C24	−158.4 (5)
C16—Fe1—C17—C18	119.0 (5)	C17—Fe1—C25—C24	169.6 (6)
C19—Fe1—C17—C18	36.7 (3)	C19—Fe1—C25—C24	−75.0 (6)
C25—Fe1—C17—C16	44.2 (10)	C18—Fe1—C25—C24	−45.8 (12)
C20—Fe1—C17—C16	−38.0 (3)	C30—N4—C26—C27	2.0 (8)
C24—Fe1—C17—C16	−158.4 (9)	Zn1—N4—C26—C27	177.5 (4)
C22—Fe1—C17—C16	122.9 (6)	N4—C26—C27—C28	−1.7 (9)
C21—Fe1—C17—C16	79.3 (6)	C26—C27—C28—C29	0.6 (9)
C23—Fe1—C17—C16	165.0 (5)	C27—C28—C29—C30	0.1 (8)
C19—Fe1—C17—C16	−82.3 (3)	C27—C28—C29—C37	−179.3 (6)
C18—Fe1—C17—C16	−119.0 (5)	C26—N4—C30—C29	−1.3 (7)
C16—C17—C18—C19	0.4 (6)	Zn1—N4—C30—C29	−177.2 (4)
Fe1—C17—C18—C19	−59.0 (4)	C26—N4—C30—C31	178.6 (5)
C16—C17—C18—Fe1	59.4 (3)	Zn1—N4—C30—C31	2.7 (6)
C25—Fe1—C18—C19	−39.1 (10)	C28—C29—C30—N4	0.2 (7)
C20—Fe1—C18—C19	37.7 (3)	C37—C29—C30—N4	179.6 (5)
C24—Fe1—C18—C19	−73.7 (5)	C28—C29—C30—C31	−179.6 (5)
C22—Fe1—C18—C19	−157.0 (5)	C37—C29—C30—C31	−0.2 (7)
C21—Fe1—C18—C19	166.5 (9)	C35—N5—C31—C32	−1.6 (7)
C23—Fe1—C18—C19	−115.1 (5)	Zn1—N5—C31—C32	173.6 (4)
C16—Fe1—C18—C19	81.9 (3)	C35—N5—C31—C30	178.9 (5)
C17—Fe1—C18—C19	119.9 (5)	Zn1—N5—C31—C30	−5.9 (5)
C25—Fe1—C18—C17	−159.0 (9)	N4—C30—C31—N5	2.7 (7)
C20—Fe1—C18—C17	−82.2 (3)	C29—C30—C31—N5	−177.4 (4)
C24—Fe1—C18—C17	166.4 (4)	N4—C30—C31—C32	−176.8 (4)
C22—Fe1—C18—C17	83.1 (6)	C29—C30—C31—C32	3.0 (7)
C21—Fe1—C18—C17	46.6 (10)	N5—C31—C32—C36	177.1 (5)
C23—Fe1—C18—C17	125.0 (5)	C30—C31—C32—C36	−3.4 (7)
C16—Fe1—C18—C17	−38.0 (3)	N5—C31—C32—C33	0.0 (8)
C19—Fe1—C18—C17	−119.9 (5)	C30—C31—C32—C33	179.5 (5)
C17—C18—C19—C20	0.2 (6)	C36—C32—C33—C34	−177.3 (6)
Fe1—C18—C19—C20	−58.4 (4)	C31—C32—C33—C34	−0.4 (8)
C17—C18—C19—Fe1	58.6 (4)	C32—C33—C34—C35	2.4 (9)
C25—Fe1—C19—C18	165.7 (5)	C31—N5—C35—C34	3.7 (8)
C20—Fe1—C19—C18	−119.9 (5)	Zn1—N5—C35—C34	−170.1 (4)
C24—Fe1—C19—C18	124.6 (5)	C33—C34—C35—N5	−4.1 (10)
C22—Fe1—C19—C18	54.7 (9)	C31—C32—C36—C37	0.9 (9)
C21—Fe1—C19—C18	−165.4 (9)	C33—C32—C36—C37	177.7 (6)
C23—Fe1—C19—C18	83.8 (5)	C32—C36—C37—C29	2.0 (9)
C16—Fe1—C19—C18	−81.7 (3)	C28—C29—C37—C36	177.0 (6)
C17—Fe1—C19—C18	−37.5 (3)	C30—C29—C37—C36	−2.4 (9)
